# Pre-pandemic brain structure and COVID-19 fear: insights into posttraumatic stress and general distress

**DOI:** 10.3389/fpsyt.2025.1643375

**Published:** 2025-12-10

**Authors:** Ting Zhang, Dongmei Wu, Yixun Tang, Guocheng Zhao, Lu Yang, Song Wang

**Affiliations:** 1 Department of Health Humanities Research Center, Sichuan Vocational College of Health and Rehabilitation, Zigong, Sichuan, China; 2 Department of Nursing, Sichuan Provincial People’s Hospital, School of Medicine, University of Electronic Science and Technology of China, Chengdu, China; 3 The Clinical Hospital of Chengdu Brain Science Institute, Ministry of Education (MOE) Key Lab for Neuroinformation, University of Electronic Science and Technology of China, Chengdu, China; 4 Nursing Department, The Sixth People’s Hospital of Chengdu, Chengdu, China; 5 Department of Radiology and Huaxi Magnetic Resonance (MR) Research Center (HMRRC), West China Hospital of Sichuan University, Chengdu, China

**Keywords:** posttraumatic stress disorder, fear, emotion, distress, brain, COVID-19

## Abstract

**Background:**

Various brain regions are implicated in fear responses to specific stimuli. While prior research has explored neural connectivity related to fear during COVID-19, gaps remain regarding the specific role of pre-pandemic brain structure in COVID-19-related fear and its impact on long-term psychological problems. This prospective longitudinal study aimed to explore the neural structural basis of individual differences in fear of COVID-19 during the peak of the COVID-19 outbreak in Chinese communities, as well as the neurobehavioral mechanisms by which this fear increased individual PTSD symptoms and general distress one year later.

**Methods:**

Preceding the COVID-19 outbreak, 115 university students from Chengdu, China, were recruited and underwent 3.0T magnetic resonance imaging scans to collect brain structural data. During the peak of the COVID-19 community outbreak, participants’ fear was assessed using the Fear of COVID-19 Scale. One year later, long-term post-traumatic stress disorder (PTSD) symptoms and general distress were measured. Associations between Pre-Pandemic Brain Structure, COVID-19 Fear, Posttraumatic Stress and General Distress were examined using Structural equation model.

**Results:**

Whole-brain multiple regression analysis identified that baseline gray matter volume (GMV) in the left (r=0.42, p<0.001) and right insulae (r=0.39, p<0.001) was positively associated with the fear of COVID-19, after adjusting for sex, age, and total GMV. Mediation analysis revealed that this fear mediated the impact of bilateral insular GMV on PTSD symptoms and general distress one year later.

**Conclusions:**

The baseline bilateral insular GMV played a pivotal role in driving the level of fear during the peak of community outbreaks of COVID-19. Additionally, fear served as a crucial mediating factor influencing the association between insular and future psychological problems. These findings could aid in identifying vulnerable populations susceptible to fear during infectious disease outbreaks like COVID-19 and provide insights into identifying target areas for mental health interventions at different stages of future outbreaks.

## Introduction

Massive disasters could lead to substantial rises in mental health problems, both in the immediate aftermath and persisting over extended durations (1, 2). COVID-19, as a natural disaster, often causes widespread mental health issues (3). It represents a novel form of traumatic event with profound mental health repercussions, including the development of post-traumatic stress disorder (PTSD) and general psychological distress (e.g., depression, anxiety, and stress) (4). Specifically, one month after the COVID-19 outbreak, the prevalence of acute PTSD symptoms among the Chinese public was 4.6% (5). After one year of the COVID-19 pandemic, the prevalence of PTSD symptoms among Chinese residents rose to 9.28% (6). In critically ill survivors, this figure is even higher (7). PTSD resulting from COVID-19 is often accompanied by generalized anxiety, depression (8), increased stigma (9), and a host of negative social issues (10). Compared to PTSD, which is characterized by psychotic features, general distress, as a more prevalent psychological health issue, has a higher detection rate during COVID-19. More than one-third of adults in the general population experience psychological distress related to COVID-19 (11, 12), and it might remain stable (13, 14). Meanwhile, a significant body of behavioral research has illustrated a reciprocal relationship between PTSD and psychological distress (15, 16). Considering the common comorbidity between post-traumatic stress and general distress, investigating their underlying neurobehavioral mechanisms could lay the groundwork for intervention strategies.

As such a behavioral influential factor for PTSD and general distress, fear is a biologically basic emotion, typically conceptualized as an adaptive yet transient state triggered by encountering a threatening stimulus (17), for example, in the face of any catastrophic, deadly pandemic (18). Particularly during the initial peak of community outbreaks of COVID-19, individuals grappled with uncertainties about their future and potential threats to their lives, making fear a prominent emotion (19, 20). In March 2020, Ahorsu et al. developed a scale for measuring fear of COVID-19 (21), catalyzing subsequent research on fear (22). It is noteworthy that individual differences in the fear of COVID-19 may exist. These variances could be associated with factors such as sex, age, level of education, understanding of COVID-19, perceptions of government pandemic response (23),individuals emotional regulation strategies (24), tolerance of uncertainty (25), cultural background (26), and others. Previous study has shown that the highest levels of fear of COVID-19 were observed in the Asian region (26). The intensity and manifestations of COVID-19 fear demonstrate significant variation across cultural contexts. A comparative study examining psychological responses to the pandemic among university students in Poland and Japan revealed that Japanese participants exhibited markedly higher levels of COVID-19 fear than their Polish counterparts. This disparity potentially stems from Japan’s geographical proximity to the pandemic’s origin and its collectivist culture’s emphasis on group harmony (27). Additional research indicates that during the Omicron variant of the COVID-19 pandemic, Chinese individuals demonstrated greater emphasis on individual absolute responsibility towards the collective and avoided becoming “troublemakers” compared to Koreans. They exhibited more protective behaviors to alleviate psychological distress such as anxiety through their inner sense of shame and fear of stigma (28).

Fear of COVID-19 has been associated with various negative psychological health issues in the general population, such as stress, anxiety, depression, sleep problems, suicidal ideation (29, 30). A meta-analysis revealed a strong positive correlation between fear of COVID-19 and post-traumatic stress (r = 0.54, n = 8,752), as well as distress symptoms (r = 0.53, n = 11,785) (31). Additionally, a longitudinal study conducted across multiple countries found that fear of COVID-19 significantly predicts the development of PTSD (32, 33). And patients diagnosed with PTSD exhibit compromised fear inhibition (34). Previous studies have shown the significant role of fear conditioning in posttraumatic stress disorder (PTSD) (35), where inadequate fear memory extinction may contribute to the onset of PTSD (36). The impact of fear on PTSD may involve neural mechanisms in brain regions such as the prefrontal cortex, hippocampus, and amygdala (37). Moreover, extensive previous studies indicated a positive correlation between fear of COVID-19 and individual distress (38–41). The fear of COVID-19 might exert its impact on distress through external factors, such as disruptions in lifestyle and leisure restrictions (42), and through psychological characteristics such as expressive suppression, cognitive reappraisal, and intolerance of uncertainty (43). A cross-global study (including Asian countries) demonstrates cultural differences in the relationship between emotion regulation strategies and mental health indicators during the COVID-19 epidemic. In individualistic cultures, individuals tend to employ strategies that maintain their authentic emotional experiences and inner states, such as cognitive reappraisal, which modifies emotional impact by altering event perception and correlates with fewer psychological issues. In collectivist cultures (Asian countries), individuals more frequently utilize expressive inhibition to maintain social harmony and group interests by concealing their emotions, particularly negative ones, which strongly correlates with anxiety and depression symptoms (44). Research indicates that prolonged suppression and concealment of these emotions may result in emotional disorders and mental health problems. A study of undergraduate nursing students in China revealed that poor emotional regulation during COVID-19 (such as using expressive suppression strategies) significantly increases psychological distress symptoms, including depression, anxiety, and PTSD -like symptoms (45). This suggests that within a collectivist cultural context, the specific emotion regulation strategies adopted by individuals significantly influence the psychological distress arising from their perception of COVID-19 risks.

The brain structures of the fear circuitry often exhibit potential overlap with other common mood disorders, such as anxiety (46). Although the pivotal role of the amygdala in fear is widely acknowledged, it does not necessarily mean that the experience of fear originates solely from this brain region. The subjective experience of fear might rely more on brain areas responsible for cognitive processes, such as the lateral and medial prefrontal cortex, parietal neocortex, and insula (47). Additionally, these brain regions may overlap with those implicated in PTSD and distress. Structural and functional MRI studies in individuals with PTSD and distress have revealed changes in brain regions, including the hippocampus, amygdala, inferior parietal lobule, and insula (48–50), suggesting a potential shared neural architecture underlying fear. Notably, ample evidence suggests the involvement of distinct brain regions and neural circuits in specific object-related fears (51). For instance, compared to snake phobia, dental phobia shows an increase in volume of the right subgenual anterior cingulate cortex, left insula, left orbitofrontal cortex, and left prefrontal cortex (52). And the brain regions involved in fear of pain include the anterior cingulate cortex, the anterior part of the posterior cingulate cortex, and the dorsomedial prefrontal cortex (53). It is worth noting that synaptic plasticity within the fear circuitry (54) underscores the necessity of investigating neural mechanisms behind specific fears. We haven’t found literature specifically addressing pre-pandemic brain structure predicting fear related to COVID-19. However, one study identified pre-pandemic neural connectivity patterns predicting heightened fear symptoms during the COVID-19 peak, with key predictors including connections such as those between the right dorsal anterior cingulate cortex and the left insula, and between the left ventromedial prefrontal cortex and the right dorsolateral prefrontal cortex, among others (55). Therefore, we have grounds to speculate that certain brain structures may predict fear of COVID-19 during the peak of community outbreaks. Furthermore, considering the predictive capacity of COVID-19 fear for PTSD and general distress in behavioral studies, along with their shared neural architecture in specific brain regions, we anticipate that these brain areas may indirectly impact long-term PTSD symptoms and general distress through the fear of COVID-19 during community outbreak peaks.

**Figure 1:**
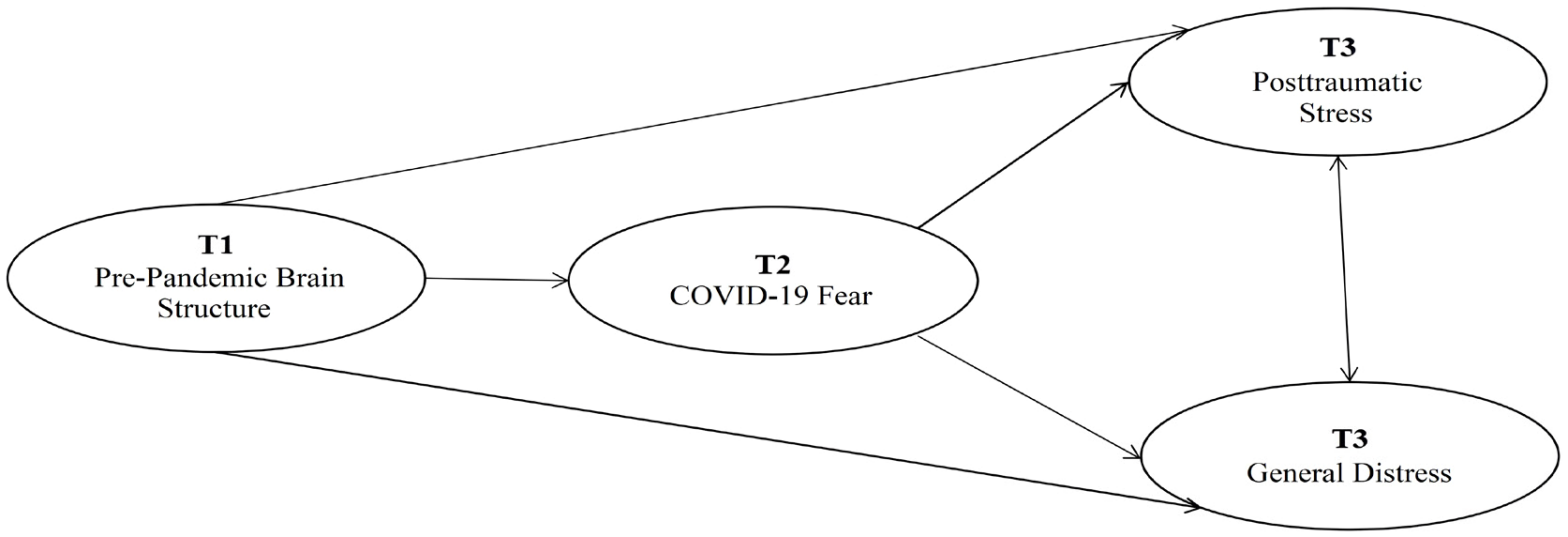
Hypothetical model diagram.

In summary, this study aims to construct a longitudinal mediation model through three follow-up measurements to investigate the effects of Pre-Pandemic Brain Structure on Posttraumatic Stress and General Distress in the third measurement (T3), as well as the mediating role of COVID-19 fear in the second measurement (T2). Assuming the model is shown in **Figure 1**.

## Methods

### Participants and procedure

A total of 151 university students from Chengdu, China, were initially recruited for this study. Ultimately, 115 participants (comprising 42.6% males, with ages ranging from 19 to 27 years) actively contributed by undergoing MRI scans and completing all scheduled questionnaires as required.

The inclusion and exclusion criteria were applied to ensure the homogeneity of the participant sample. Participants meeting the following inclusion criteria were eligible for the study: (1) right-handedness based on the Edinburgh Handedness Inventory (56); (2) native speakers of Mandarin Chinese; (3) provided informed consent to participate. Conversely, individuals with a medical history of neurological or psychiatric disorders, sleep disorders, or MRI contraindications were excluded.

Following the recruitment of participants, the study procedures were carried out as follows. Participants underwent structural brain MRI scans and basic sociodemographic and behavioral measurements between October 13, 2019, and January 19, 2020 (T1), pre-dating the onset of the COVID-19 pandemic. Subsequently, during the peak of the community outbreak of COVID-19 (57), participants completed measurements of fear of COVID-19 from April 1 to April 30. Approximately one year later, between March 1, 2021, and April 30, 2021 (T3), during the later stage of the COVID-19 pandemic peak, participants underwent measurements of posttraumatic stress and general distress. Ethical approval was obtained from the Medical Research Ethics Committee of West China Hospital of Sichuan University prior to the commencement of the study.

### Behavioral measures

#### The fear of COVID-19

At T2, the Fear of COVID-19 Scale (FCS-19), developed by Ahorsu et al. in 2020 (21), was utilized to assess the participants’ fear of COVID-19. Consisting of seven items, it demonstrates a stable one-dimensional structure. Sample items include “I am most afraid of coronavirus-19” and “My heart races or palpitates when I think about getting coronavirus-19.” Utilizing the Likert 5-point scoring method, responses range from 1 for “Strongly Disagree” to 5 for “Strongly Agree,” yielding a total score range of 7 to 35, with higher scores indicating greater fear of COVID-19. The original scale’s item factor loadings range from 0.66 to 0.74, demonstrating good internal consistency with a Cronbach’s alpha coefficient of 0.82. Test-retest reliability is satisfactory, with an Intraclass Correlation Coefficient (ICC) of 0.72, and the scale exhibits measurement invariance across genders and age groups. Widely adopted globally (58), the scale also shows robust internal consistency among Chinese populations, with an alpha coefficient of 0.92 (59).

#### Posttraumatic stress

In this study, participants used the Posttraumatic Stress Disorder Checklist (PCL-5) from the Diagnostic and Statistical Manual of Mental Disorders, Fifth Edition (DSM-5) (60), to self-assess posttraumatic stress at T3. The checklist comprises four subscales (intrusion, avoidance, negative alteration in cognitions and mood, and arousal) with a total of 20 items, rated on a scale from 1 (not at all) to 5 (extremely), yielding a total score range of 20-100. The reliability and validity of this instrument have been extensively investigated across diverse populations. During the COVID-19 pandemic in China, the PCL-5 showed good reliability and validity, with a total score Cronbach’s alpha of 0.91, subscale internal consistency reliability ranging from 0.74 to 0.90, and all items having factor loadings above 0.7 on their respective subscales (61).

#### General distress

This study employed the Depression, Anxiety, and Stress Scale-21 (DASS-21) (62) to measure the general distress of participants at T3. The scale comprises 21 items, each rated from 1 (never) to 4 (always), with a total score ranging from 21 to 84. Higher scores indicate higher levels of overall distress. Widely utilized globally, the scale has demonstrated good internal consistency among Chinese university students, with an internal consistency coefficient of 0.92 for the total DASS score (63), and it has been widely employed during the COVID-19 pandemic to measure general distress among populations (64, 65).

#### Other controlling factors

To control for potential confounding factors that may affect the links between the fear of COVID-19, posttraumatic stress, General distress, and identified gray matter volume (GMV) in the brain, at T1 we administered the Subjective Socioeconomic Status Scale (SSS) (66) to assess participants’ socioeconomic status and used the Self-Rating Life Events Checklist (SRLEC) to evaluate the frequency and impact of stressful life events experienced by participants over the past year (67, 68). Both measures demonstrated satisfactory internal reliability (69).

### MRI examination and preprocessing

The whole-brain structural magnetic resonance imaging (S-fMRI) examination was conducted using a 3.0 T Siemens-Trio Erlangen MRI scanner equipped with a 12-channel head coil. To minimize scanner noise and head movement, participants were provided with earplugs and foam padding. Each participant underwent scanning with a magnetization-prepared rapid gradient-echo sequence. The imaging parameters were set as follows: an echo time of 2.26 ms, an inversion time of 900 ms, a repetition time of 1,900 ms, a voxel size of 1×1×1 mm^3, 176 sagittal slices with a thickness of 1 mm, a field of view of 240×240 mm^2, a matrix size of 256×256, and a flip angle of 9°.

Prior to MRI scan preprocessing, an experienced medical radiologist inspected the images to identify any visible movement artifacts or anatomical abnormalities. The inspected images then underwent preprocessing using the Statistical Parametric Mapping program (SPM12; Wellcome Department of Cognitive Neurology, London, UK; http://www.fil.ion.ucl.ac.uk/spm/) following the standard procedures outlined in the VBM Tutorial (70). These procedures included: (1) manual reorientation to align the origin of the images with the anterior–posterior commissure line; (2) segmentation into white matter, gray matter, and cerebrospinal fluid; (3) alignment and resampling of gray matter images to 1.5 × 1.5 × 1.5 mm3 and normalization to Montreal Neurological Institute (MNI) space using the Diffeomorphic Anatomical Registration Through Exponentiated Lie algebra (71); and (4) modulation of gray matter values in each voxel with Jacobian determinants, followed by smoothing with an 8-mm full width at half maximum (FWHM) Gaussian kernel. Subsequently, the images depicting the regional GMV were obtained and used for further analysis.

### Statistical analyses

Because we used the online questionnaire method to collect behavioral data, which required completion of all items before submission, there was no missing data. Thus, all analyses were conducted on the complete dataset.

### Whole-brain regression analysis

A whole-brain multiple regression analysis was conducted to identify neural structures associated with variations in fear of COVID-19. The dependent variable was the GMV of each voxel across the entire brain, while the independent variable was the fear of COVID-19 score. To address potential confounding factors, sex, age, and total GMV were included as covariates in the regression model. An absolute threshold masking of 0.2 was applied to minimize edge effects between white and gray matter (71). Correction for multiple comparisons was performed using a voxel wise threshold of P < 0.001 and a cluster threshold of P < 0.05, employing the Gaussian random field theory correction method (72).

### Correlation and mediation analysis

Using Pearson correlation analysis, we examined the correlation between the GMV of identified brain regions and posttraumatic stress and general distress. To examine the hypothesis that the fear of COVID-19 at Time 2 may act as a mediator in the relationship between GMV at T1 and posttraumatic stress/general distress at Time 3, we conducted Structural Equation Model (SEM) analyses using maximum likelihood estimation in AMOS software (version 22.0) (IBM Corporation, Armonk, NY, USA). In this model, we considered the fear of COVID-19 score as the mediator, GMV (previously associated with COVID-19 fear) as the independent variable, and PCL-5 and DASS-21 scores as the dependent variables. Additionally, we included sex, age, and total GMV as control variables. The model fit was evaluated using a combination of indices, with acceptable fit defined as follows: χ²/df < 5, comparative fit index (CFI)/non-normal fit index (NNFI) > 0.9, and root mean square error of approximation (RMSEA)/standardized root mean square residual (SRMR) < 0.05 (73). Furthermore, to ascertain the statistical significance of indirect and direct effects, we employed a bias-corrected bootstrapping procedure generating 5000 surrogate datasets with 95% confidence intervals (CIs) (74). Standardized regression coefficients (βvalues) were used to estimate the path coefficients in the model.

## Results

### Demographic and behavioral characteristics

**Table 1** presents the means, standard deviations, ranges, and bivariate correlations of the behavioral variables. The fear of COVID-19 was not linked with age (r = -0.07, p > 0.05), SSS (r = -0.07, p > 0.05), and the number of other stressful events in the past years (r = 0.13, p > 0.05), but linked with sex (r = 0.25, p < 0.01; male 0, female 1) and the impact of other stressful events in the past years (r = 0.19, p < 0.01). As expected, there was a positive association of fear of COVID-19 with PTSD symptoms (r = 0.42, p < 0.001) and general distress (r = 0.38, p < 0.001). Importantly, this association remained even after adjusting for age, sex, SSS and the number and impact of other stressful events in the past years (for the PTSD symptoms: partial r = 0.40, p < 0.001; for the general distress: partial r = 0.36, p < 0.001). We then investigated the structural brain markers of fear of COVID-19 and their relations to PTSD symptoms and general distress.

**Table 1:** Descriptive statistics of various variables and associations between the variables(N=115).

Measure (Time)	Mean ± SD	Range	1	2	3	4	5	6	7
1. Sexa (T1)	–	–	–						
2. Age (years) (T1)	22.37 ± 2.08	19-27	-0.10	–					
3. SSS (T1)	9.89 ± 2.98	3-18	0.02	0.05	–				
4. SRLEC-Number (T1)	12.23 ± 5.78	1-27	-0.04	-0.06	-0.22*	–			
5. SRLEC-Impact (T1)	28.11 ± 16.66	2-77	0.04	-0.02	-0.20*	0.92*	–		
6. FCS-19 (T2)	9.79 ± 3.51	7-22	0.25**	-0.07	-0.07	0.13	0.19**	–	
7. PCL-5 (T3)	24.85 ± 7.32	20-59	0.05	-0.05	-0.17	0.31***	0.28**	0.42***	–
8. DASS-21 (T3)	35.77 ± 10.51	21-59	-0.01	0.05	-0.21*	0.22*	0.27*	0.38***	0.40***

SD, standard deviation; SSS, subjective socioeconomic status; SRLEC, Self-Rating Life Events Checklist; FCS-19, Fear of COVID-19 Scale; PCL-5, Posttraumatic Stress Disorder Checklist for DSM-5; DASS-21, Depression, Anxiety, and Stress Scale-21. Timepoints: T1, October 2019 to January 2020; T2, April 2020; T3, March to April 2021. a Male, 0; Female, 1. ***p < 0.001; **p < 0.01; *p < 0.05.

### GMV linked to fear of COVID-19

**Figure 2:**
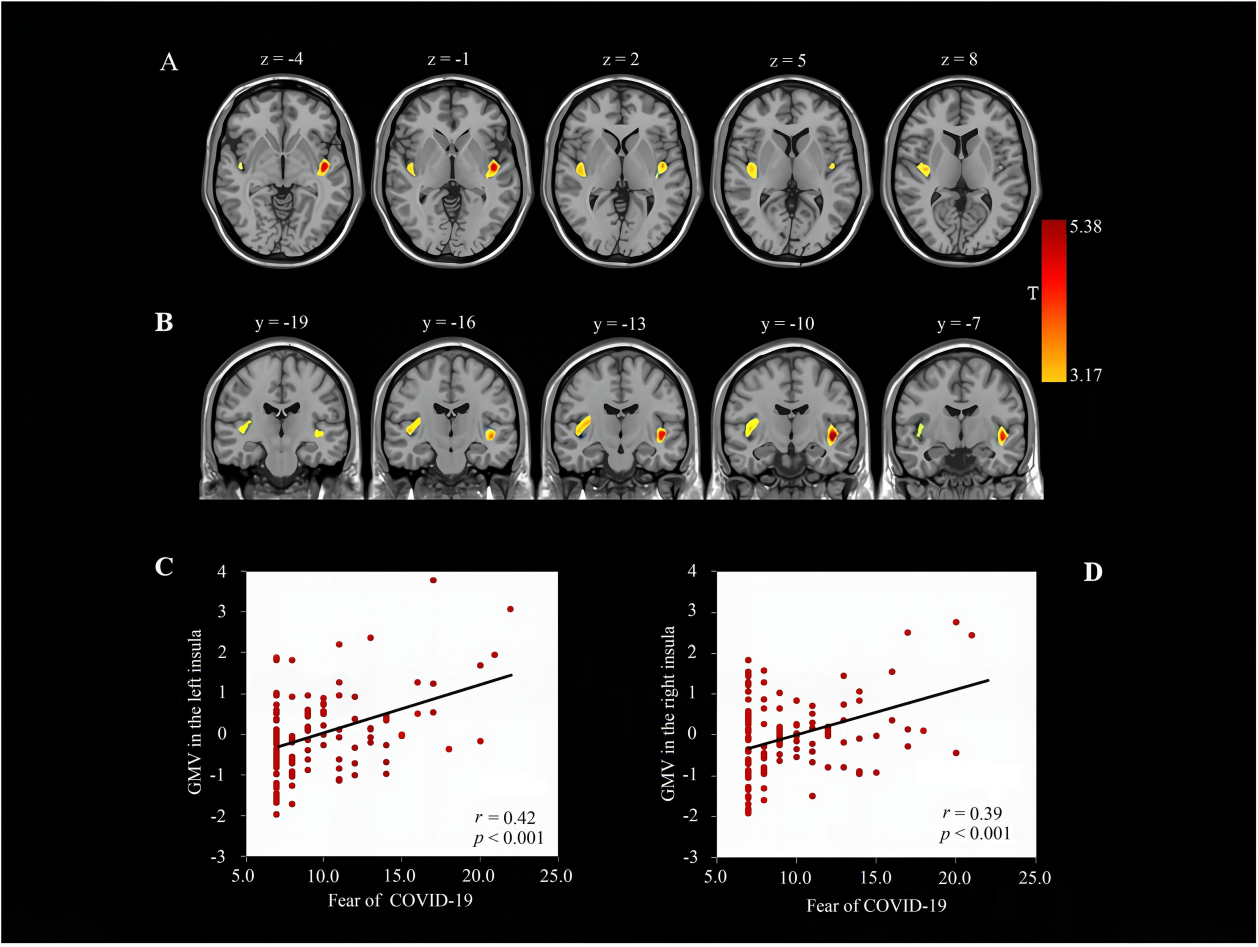
Brain regions linked to fear of COVID-19.Brain images show that fear of COVID-19 is positively linked with GMV in the left insula and right insula **(A, B)** after adjusting for sex, age, and total gray matter volume (GMV) (the color bar represents the strength of the positive correlation). Scatter plots depict the correlation between fear of COVID-19 and GMV in the left insula **(C)** and right insula **(D)**. The scores on the y-axis represent the standardized residuals of the left/right insula GMV values after regressing out sex, age, and total GMV; the scores on the x-axis represent the raw scores of Fear of COVID-19 Scale.

Following adjustments for sex, age, and total GMV, whole-brain multiple regression analyses were conducted to detect specific brain regions closely associated with fear of COVID-19. The results indicated a positive correlation between scores on the Fear of COVID-19 Scale and GMV in the bilateral insular regions (see **Table 2**, **Figure 2**). Furthermore, no other clusters significantly correlated with fear of COVID-19 were identified. Given the high correlation between GMV values in the bilateral insular regions (r = 0.82, p < 0.001), we used the mean of the bilateral insular GMV as the index of brain feature linked with fear of COVID-19 in the subsequent analyses.

**Table 2:** Correlation analysis of brain regions associated with fear of COVID-19.

Region	BA	Peak MNI coordinate			Peak T	Cluster size (voxels)
		x	y	z	Score	
Left insula	13/22	-43.5	-10.5	-4.5	5.38	597
Right insula	13/22	36	-13.5	9	4.19	550

BA, Brodmann area; MNI, Montreal Neurological Institute. Multiple corrections for the resulting map were inferred using Gaussian random field theory (p < 0.05 at the cluster level combined with p < 0.001 at the voxel level).

### Bilateral insula linked fear of COVID-19 to posttraumatic stress and general distress

After identifying brain structures linked with fear of COVID-19, we further explored the underlying neurobehavioral mechanism of how fear of COVID-19 affected PTSD symptoms and general distress. To this end, we first performed correlation analyses and showed that the bilateral insular GMV linked with fear of COVID-19 was also correlated with PTSD symptoms (r = 0.27, p < 0.01) and general distress (r = 0.22, p < 0.05) after adjusting for sex, age and total GMV. This correlation persisted (for the PTSD symptoms: r = 0.26, p < 0.01; for the general distress, r = 0.21, p < 0.05) even after additionally controlling for SSS and the number and impact of other stressful events in the past years.

**Figure 3:**
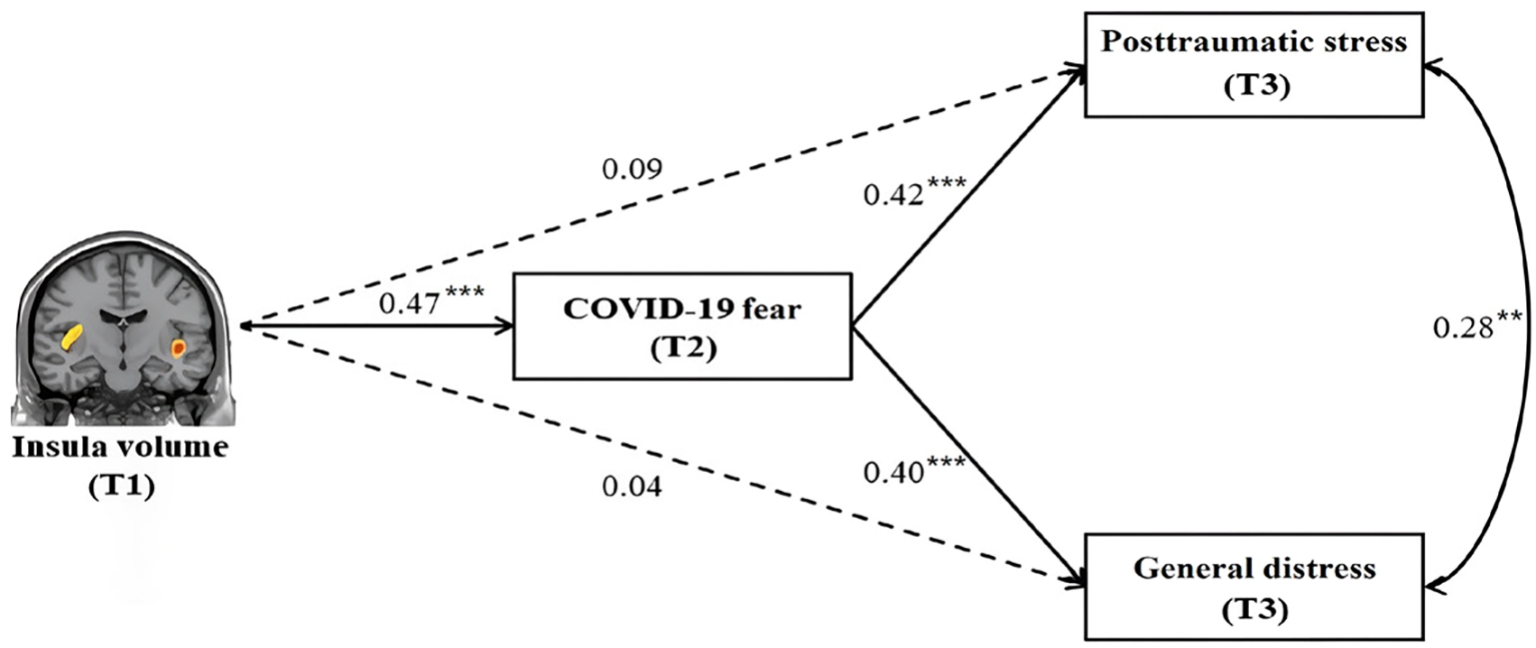
Structural equation modeling results of insula volume, COVID-19 fear and posttraumatic stress symptoms and general distress. Sex, age and total GMV are controlled for in the model and standardized estimates are presented. The symbols ** and *** represent the following levels of statistical significance, as is standard in our field: ***p < 0.001; **p < 0.01.

We then performed a SEM analysis to test the possible mediating effect of COVID-19 fear on the link of insular GMV with PTSD symptoms and general distress, with age, sex and total GMV as the covariates. The results revealed a good fit (χ2 [2] = 1.08, p = 0.583; NNFI = 0.98, CFI = 1.00, RMSEA < 0.001) for the proposed model. The pathway results showed that increased insular GMV was associated with greater fear of COVID-19 (β = 0.47, p < 0.001); in turn, greater fear of COVID-19 was associated with more PTSD symptoms (β = 0.42, p < 0.001) and general distress (β = 0.40, p < 0.001) (see **Figure 3**). Crucially, a significant mediating effect was observed from increased insular GMV to more PTSD symptoms (indirect effect = 0.197, 95% CI = [0.076, 0.322], p < 0.001) and general distress (indirect effect = 0.186, 95% CI = [0.069, 0.320], p < 0.001) via greater fear of COVID-19. This effect remained even after additionally adjusting for SSS and the number and impact of other stressful events in the past years (for the PTSD symptoms: indirect effect = 0.193, 95% CI = [0.087, 0.309], p < 0.001; for the general distress: indirect effect = 0.171, 95% CI = [0.065, 0.292], p < 0.001). In brief, fear of COVID-19 might be a mediator in the association of insular GMV with PTSD symptoms and general distress.

## Discussion

This prospective longitudinal study aimed to explore the neural structural basis of individual differences in fear of COVID-19 during the peak of the COVID-19 outbreak in Chinese communities, as well as the neurobehavioral mechanisms by which this fear increased individual PTSD symptoms and general distress one year later. Data were obtained through structural brain imaging before the COVID-19 outbreak, behavioral assessments during the peak of the COVID-19 community outbreak, and one year later. Whole-brain multiple regression analysis and mediation analysis revealed that increased GMV in the bilateral insular cortex in baseline was associated with heightened fear of COVID-19 during the peak of the outbreak. Additionally, this fear mediated the impact of bilateral insular GMV on PTSD symptoms and general distress one year later. Our findings provided new evidence for the structural neural basis of fear of COVID-19 and offer novel neurobehavioral pathways for mitigating the long-term psychological effects of COVID-19 on mental health issues (PTSD symptoms and general distress).

Infectious disease pandemics could cause significant trauma, potentially resulting in PTSD and enduring psychological distress. Moreover, PTSD and psychological distress may co-occur and share similar risk factors such as the degree of trauma exposure, isolation and quarantine, social inequalities, and job responsibilities (75). Our findings corroborated that fear of COVID-19 during the peak of community outbreaks could predict PTSD symptoms and general distress one year later. Additionally, this association remained unaffected by sex, age, total GMV, subjective socioeconomic status and life events. This aligns with previous literature emphasizing the predictive role of fear in PTSD and general distress (32, 33). As is well known, cultural background factors play an important role in shaping emotional responses and risk perception. Research indicates that countries with collectivist cultures, particularly China, emphasize individual emotional restraint and strengthen avoidance strategies (such as the culture of tolerance) to maintain social harmony and group interests (44). This approach may deplete cognitive resources and diminish individuals’ ability to utilize cognitive reappraisal and other strategies, resulting in emotional regulation difficulties and ultimately perpetuating fear as PTSD symptoms or general psychological distress. Difficulty in emotion regulation likely represents the key psychological mechanism connecting COVID-19 fear and long-term symptoms in this study.

PTSD could be considered a condition characterized by dysregulation of fear processes. The effectiveness of exposure-based therapy and cognitive processing therapy in treating PTSD by reducing fear through “extinction” learning based on Pavlovian conditioning reaffirms the relationship between fear and PTSD (76). Norrholm et al.’s study found that high fear levels could predict PTSD symptoms, suggesting that measuring fear load may aid in comparing specific fears and their impact on psychological issues (77). The pathways through which fear impacts PTSD might be associated with fear conditioning dysfunction, failure of trauma-related fear memory extinction, and fear reconsolidation (35). For more common general distress, which includes stress, anxiety, and depression, it significantly impacts the psychological well-being of both current and future populations (78). The fear of COVID-19 has consistently shown incremental validity in explaining significant indicators of psychological distress (79). Previous study has shown that in populations experiencing broad distress, there might be dysregulation in fear circuits, but the causal relationship remains unclear (80). Our study revealed that fear of COVID-19 at the peak of the outbreak could positively predict individuals’ general distress one year later. These findings reveal that interventions targeting fear during the peak of major infectious disease outbreaks may effectively reduce the development of long-term psychiatric disorders and common psychological issues in the population. They provide valuable insights for the timing of psychological interventions at different stages of the human response to high-threat lethal infectious diseases.

Our study findings indicated that individuals with larger insular GMV experienced greater fear of COVID-19 during peak outbreaks in COVID-19 communities. This is consistent with previous findings from functional magnetic resonance imaging studies, which showed heightened insular responses associated with higher levels of fear (81). The insula played a pivotal role in contributing to the processing of cognitive, emotional, and primal responses to fear stimuli (82). Different neuronal populations within the insula might encode various processes of conditioned fear (83). The insular cortex was considered responsible for integrating consciousness (including cognition, emotion, and bodily states), and had also been suggested to facilitate higher-level appraisal and anticipatory processes relevant to the conscious perception of fear (84). The insula was involved in individuals’ responses to unpredictable threats (85). Previous study indicated that the insula exhibited sensitivity to significant environmental stimuli and suggested that an enlargement in its GMV could potentially bolster an individual’s capacity to recognize fear even at low levels of awareness (86). This ability might be linked to its role in directing attention towards significant stimuli in the environment, and increased insula GMV was associated with prioritized allocation of attentional resources to threatening stimuli (87). Another investigation into GMV among university students revealed a positive correlation between insula volume and emotional monitoring abilities (88), which might also contribute to heightened fear. Furthermore, the positive correlation observed between insula grey matter volume and individual social network metrics might serve as a contributing factor (89). Amid the COVID-19 pandemic, individuals might face increased risk of COVID-19 exposure due to complex social networks, thus indirectly heightening fear of COVID-19.

Our study also found that larger insular GMV at T1 were associated with more PTSD symptoms and general distress one year later. In structural and functional MRI studies related to the pathophysiology of PTSD, the insula has been demonstrated to exhibit high reproducibility (49). A meta-analysis of voxel-based morphometry studies found a positive correlation between grey matter in the left insula and PTSD symptoms (90). Increased activation of the insular cortex may heighten individuals’ responsiveness to emotional processing (91), exacerbating psychological distress such as stress, discomfort, and avoidance induced by uncertainty (92). And the link between the insular cortex and psychological distress might involve dysregulation of corticotropin-releasing factor (CRF), CRF receptor 1, and cannabinoid receptor 1 (93). Therapeutic interventions targeting the insular cortex, such as transcranial magnetic stimulation (94), selective serotonin reuptake inhibitors, and cognitive-behavioral therapy (95), have demonstrated effectiveness in reducing stress, anxiety, and negative emotions, thus underscoring the pivotal role of the insular cortex in the onset or progression of PTSD symptoms and general distress disorders. Furthermore, our study findings also revealed that larger gray matter volumes in the insula at T1 could indirectly influence higher levels of post-traumatic stress disorder (PTSD) symptoms and general distress one year later through higher levels of fear of COVID-19 at T2. The insular cortex might contribute to the development of long-term psychological disorders through fear generalization (96). Studies suggested its involvement in modulating the frequency of intrusive memories through fear acquisition and extinction (97), as well as facilitating the progression of long-term PTSD symptoms and general distress through mechanisms such as re-experiencing (98) and the associated memory extinction deficits (99). Our findings suggest that intervening on fear during peak COVID-19 outbreaks could reduce long-term PTSD symptoms and psychological distress, providing insights into identifying target areas for mental health interventions at different stages of future outbreaks.

This study has several limitations that warrant attention. Firstly, there was an undeniable presence of social desirability bias in self-reported questionnaire surveys. Future research should incorporate clinical interview style assessments or objective physiological indicators as supplements to enhance data robustness and reduce such biases. Secondly, our results detected changes in insular GMV, with no positive results for other brain regions such as the hippocampus and amygdala, which have been reported to be linked with fear-related processing (100). This might be attributed to the fact that the samples came from various sources, and only GMV was measured, neglecting other brain structures such as topology and white matter. It is suggested that future research can integrate multimodal neuroimaging data, such as resting state functional connectivity, task state fMRI, and cortical thickness analysis, particularly examining fear-related neural networks including the amygdala insula prefrontal cortex, in order to more deeply reveal the neurodynamic mechanisms of fear processing and subsequent development of psychiatric symptoms. Thirdly, our sample is only composed of Chinese university students, which has an advantage in sample homogeneity but limits the generalizability of the research results to other age groups, cultural backgrounds, or broader community populations. Additionally, we do not explore the potential influence of gender or cultural factors on COVID-19 fear and psychological distress. Future research should aim to conduct in a sample with greater demographic diversity, including groups of different ages, genders, occupations, and cultural backgrounds, and engage in multi center collaboration to validate the generalizability of the findings of this study. Finally, although MRI data collection was conducted before the outbreak of COVID-19, it was impossible to check whether individuals were infected with COVID-19 before the outbreak. Although the possibility of infection is very low, the fact remains that COVID-19 may affect the brain (101). Furthermore, subsequent studies should consider the possibility of asymptomatic infections or other unmeasured neurological confounding factors.

In conclusion, our study provides novel evidence of the structural neural influences on fear during the peak of the COVID-19 community outbreak, revealing the potential critical role of bilateral insular GMV in driving fear levels related to COVID-19. Moreover, we found that COVID-19 fear might serve as a significant mediating factor influencing the relationship between the insular cortex and the development of future PTSD symptoms and general distress. These findings could aid in identifying vulnerable populations susceptible to fear during infectious disease (like COVID-19) outbreaks and guide targeted interventions. Furthermore, they inform the selection of brain regions for interventions [e.g., repetitive transcranial magnetic stimulation (102) targeting long-term mental and psychological issues associated with COVID-19, thereby assisting in mitigating the prevalence of long-term psychological issues stemming from COVID-19.

## Data Availability

The original contributions presented in the study are included in the article/supplementary material. Further inquiries can be directed to the corresponding author.
